# Reconceptualizing autonomic function testing in migraine: a systematic review and meta-analysis

**DOI:** 10.1186/s10194-024-01758-7

**Published:** 2024-04-10

**Authors:** Antun R. Pavelić, Karin Zebenholzer, Christian Wöber

**Affiliations:** 1 Department of Neurology, University Hospital Tulln, Alter Ziegelweg 10, Tulln, 3430 Austria; 2 Karl Landsteiner University of Health Sciences, Dr. Karl-Dorrek-Straße 30, 3500, Krems, Austria; 3 Department of Neurology, Medical University of Vienna, Währinger Gürtel 18-20, Vienna, 1090 Austria; 4 Medical University of Vienna Comprehensive Center for Clinical Neurosciences & Mental Health, Vienna, 1090 Austria

**Keywords:** Migraine, Autonomic nervous system testing, Deep breathing, Valsalva manoeuvre, Orthostatic, Isometric challenge, Parasympathetic activity, Sympathetic activity

## Abstract

**Background:**

Autonomic nervous system (ANS) testing has aided in our ability to evaluate autonomic dysfunction in migraine patients. We reviewed the literature in multiple databases which investigate ANS function in migraine patients and healthy subjects.

**Methods:**

This systematic review and meta-analysis examined the respective deep breathing, Valsalva manoeuvre, orthostatic and isometric challenge results, using the Preferred Reporting Items for Systematic Reviews and Meta-Analyses (PRISMA) and Meta-analyses of Observational Studies in Epidemiology (MOOSE) statements.

**Results:**

Seven articles met all inclusion criteria. Fixed-effects meta-analysis showed migraine patients (*n* = 424), collectively, had lower interictal autonomic test results compared with healthy controls (*n* = 268). In detail, this was true for the standardized mean difference (g) of deep breathing (g= -0.32; 95% confidence interval (CI) -0.48, -0.16), orthostatic challenge (g= -0.28; 95% CI -0.44, -0.13) and isometric challenge (g= -0.55; 95% CI -0.71, -0.39) and for the difference of means (MD) of the Valsalva ratio (MD = -0.17; 95% CI -0.23, -0.10).

**Conclusions:**

Interictal ANS dysfunction can be identified in migraine patients when compared to healthy controls. These findings indicate the importance to evaluate ANS function in migraine patients - especially, as migraine-specific prophylactic therapies (such as anti-calcitonin gene-related peptide (CGRP) antibodies) may affect the function of the ANS.

## Introduction

The relation between autonomic nervous system (ANS) dysfunction and common headache disorders (including migraine [1–3], cluster headaches [4] and tension-type headaches [5, 6]) has been widely documented. In migraine, a plethora of autonomic symptoms precedes, accompanies and outlasts the headache attacks. These symptoms include, but are not limited to nausea, vomiting, hyperhidrosis, pallor, palpitations, and light-headedness and make an attack that much more intolerable [7, 8]. An additional clinical significance of ANS dysfunction is the observed increased probability of major cardiovascular disease (CVD - hazard ratio (HR) 1.50, 95% confidence interval (CI) 1.33–1.69), myocardial infarction (odds ratio (OR) 2.2, 95% CI 1.7–2.8), ischemic stroke (OR 1.5, 95% CI 1.2–2.1), and death due to ischemic CVD (HR 1.37, CI 1.02–1.83) shown in patients suffering from migraine with and without auras [9–12]. Schürks and colleagues found that migraine is associated with a twofold increased risk of ischemic stroke, apparent only among people who have migraine with aura [13]. Thus, to expand on the argument made by Koenig et al. [9], it is not only important to understand the role of vagally mediated heart rate variability (HRV), but to also better understand overall ANS function among migraine patients and the relationship with cardio- and cerebrovascular comorbidities, using standardized investigations of the ANS.

Research offered molecular explanations for the variety of symptoms seen in migraine patients. One such explanation is the CGRP. The 37-amino acid peptide is a potent vasodilator and plays diverse roles in the human body, influencing blood pressure regulation, angiogenesis, sepsis, arthritis, inflammation and migraine [14–19]. Furthermore, in the central nervous system (CNS), CGRP has been shown to be active in the hippocampus, sets in motion other neuroprotective processes and acts on other brain cells (i.e. astrocytes or oligodendrocytes) [20]. Conversely it seems to also have an antidepressive effect [20] and to facilitate the excitotoxic death of hippocampal neurons in a kainic acid seizure model [21]. Anti-CGRP substances proved effective in providing relief to migraine patients; however, the long-term effects of CGRP modulation are only now beginning to be thoroughly described [22]. To support this argument, Tringali and Navarra expressed valid concerns in their review, that further long-term observations are required to examine the effects of CGRP-inhibition, as it pertains to autonomic function [23]. Additionally, clinicians currently have no objective method, with which to evaluate which patients stand to benefit from CGRP modulation.

Researchers investigated ANS function related to migraine and headache disorders since the 1950s. Much of the earlier work, investigating ANS function/dysfunction in migraine patients, was based on the autonomic theory. This idea postulated that much of the pathogenic migraine process could be attributed to the increase in noradrenaline from the nerve endings of the affected blood vessels [24]. The theory has since been disproven. The resulting ANS function research, however, reported a vast variety of results. Most studies showed reduced sympathetic function in migraine patients; while others reported increased sympathetic function; others still, showed normal sympathetic function. Likewise, the majority of studies reported normal parasympathetic cardiovagal function, while some reported decreased parasympathetic function [15]. Miglis goes on to describe the variety of methodologies these conclusions were derived from [15]; ultimately illustrating the need for consistent, standardized testing of the ANS in migraine studies.

In 1985, Ewing and his colleagues suggested a series of tests - which would become the standard for ANS function testing today [25–28]. This series comprises of the deep breathing, Valsalva manoeuvre, orthostatic challenge, and isometric challenge tests. From these, a variety of values can be derived, characterizing autonomic function. Cumulatively, the composite autonomic scoring scale (CASS) combines cardiovagal, sympathetic adrenergic and sudomotor function results into a single score, enabling clinicians to diagnose and monitor disease progression [26, 28].

Research using standardized ANS testing has aided to evaluate autonomic migraine symptoms – however, there currently exists neither an aggregated, nor a standard set of values, to provide diagnostic or therapeutic evaluation in the clinical or research setting. Koenig and colleagues conducted a meta-analysis of the vagally mediated HRV results in migraine patients versus healthy controls [9]; meanwhile, Lee and her colleagues conducted a meta-analysis of the electrocardiographic values between the two populations [29]. Both articles reported differences between migraine patients and healthy controls in their respective investigated parameters. In contrast, there currently exist no meta-analyses which summarize ANS function data gathered using the standardized ANS testing protocol [28]. To assess current knowledge, we performed a systematic review and meta-analysis of studies comparing ANS function in migraine patients and healthy subjects; focusing on articles which most closely matched the latest standard autonomic testing protocol, described by Novak in 2011 [28].

## Methods

### Systematic literature search

**Fig. 1:**
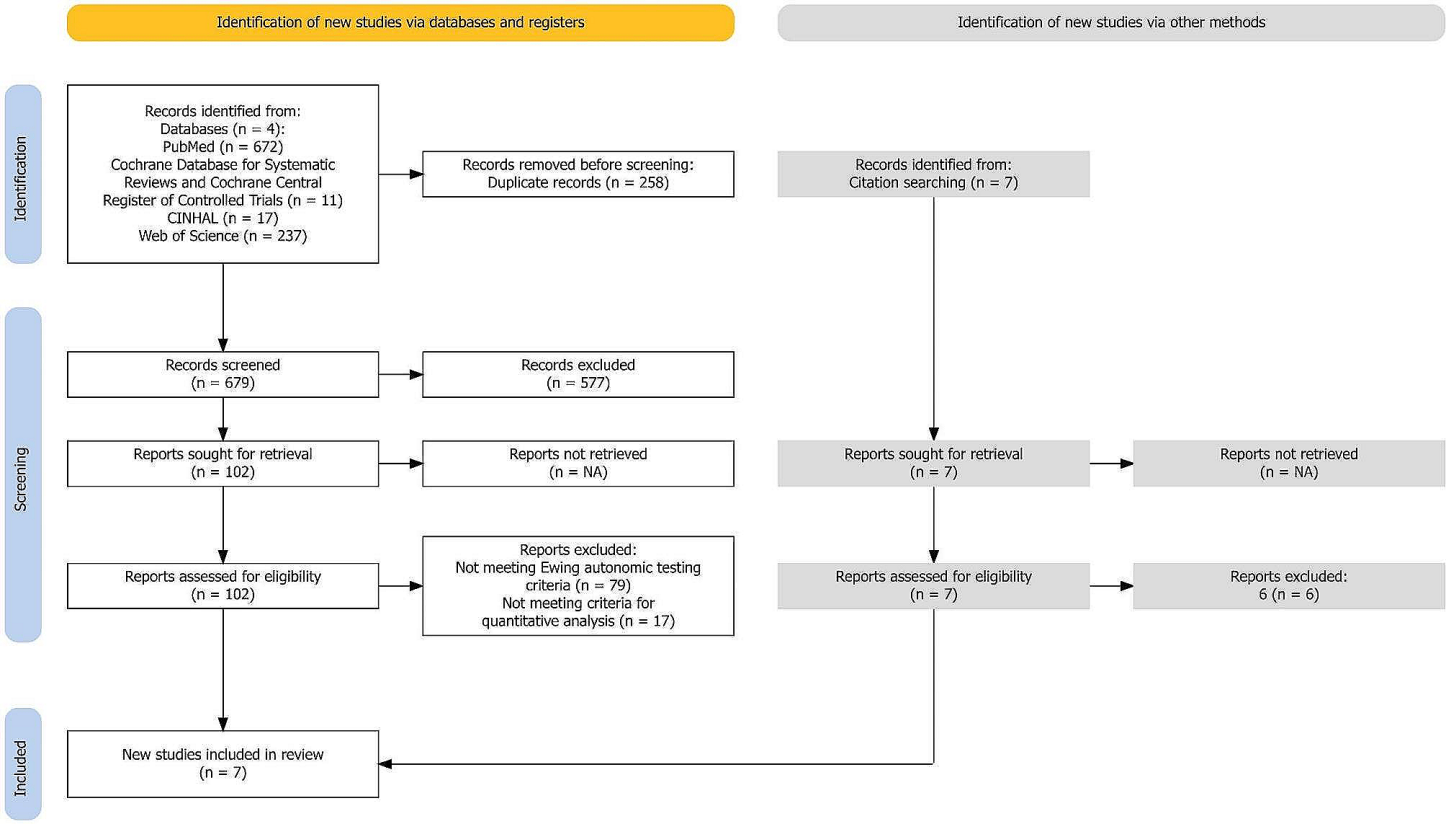
PRISMA Systematic literature search flowchart

We conducted a systematic literature search, according to the Preferred Reporting Items for Systematic Reviews and Meta-Analyses (PRISMA) and Meta-analysis of Observational in Epidemiology Studies (MOOSE) statements [30, 31]. (Fig. 1) Experienced neurologists specialising in headache disorders – one of these authors experienced in ANS function testing – conducted the search and statistical analysis. We searched PubMed library, Cochrane Database for Systematic Reviews and Cochrane Central Register of Controlled Trials, Cumulative Index to Nursing and Allied Health Literature (CINHAL) and Web of Science for the terms “autonomic testing” OR “autonomic function” AND “migraine” NOT “review”. (Appendix A). The analysis included results up to 21 November 2023.

Papers included were original cohort studies, case reports or trials of clinical interventions and non-clinical interventions; in addition, we searched the reference lists of the included studies; reviews and systematic analyses were excluded from final analysis. After removing duplicates, we scanned abstracts, based on the following inclusion criteria. Studies had to be in English; be available in full text; include human subjects; provide demographic data; apply current or earlier diagnosis criteria for migraine without and with aura (International Classification of Headache Disorders (ICHD) editions II or III [32, 33]; Classification and diagnostic criteria for headache disorders, cranial neuralgias and facial pain, first edition [34]; common or classic migraine, according to the Ad Hoc Committee for classification of migraine (AHC-CoH) [35]); and use standardized ANS testing method. To guarantee maximum consistency we selected four ANS tests initially suggested by Ewing [25] (deep breathing, Valsalva manoeuvre, orthostatic challenge, isometric challenge) which most closely resembled the ANS function investigations in the standard, internationally-accepted sequence of autonomic testing used [28, 36–38]. This battery of tests has evolved since 1985 [25], removing some tests which became clinically redundant – evaluating, for example, sympathetic function twice or simply requiring extra equipment to be purchased (e.g. dynamometer). As such, articles published before 2011, generally employed the isometric challenge test; however later studies (accepted after 2011) no longer relied on all suggested tests.

Inclusion of a study also required, that the results of deep breathing, Valsalva manoeuvre, orthostatic challenge and isometric challenge had to be given in both migraine patients and healthy controls; and the average score of the investigated methods and the standard deviation (or standard error of mean) must have been made available either in the final publication or upon request from the corresponding author. We deemed articles with missing and/or unattainable data as not having met the inclusion criteria.

### Autonomic function tests

The deep breathing test examines cardiovagal (parasympathetic) function. Cardiac responses to deep breathing are mediated by the vagal nerve, which are represented as changes in instant heart rate (also called respiratory mediated HRV). These changes are best seen by deeply inhaling at a paced rate of six breaths/minute and measuring the R-R-Interval (RRI) changes (i.e., the amplitude of the beat-to-beat variation with respiration, standard deviation of the RRI, the mean square successive difference, the expiratory-inspiratory ratio (E: I ratio), and the mean circular resultant). The observed beat-to-beat variation represents vagal input; and thus, measurement of the RRI allows to evaluate cardiovagal – parasympathetic – function [26–28, 39].

The Valsalva manoeuvre evaluates the subject’s sympathetic adrenergic functions and the cardiovagal functions. Sustained forced expiration against resistance causes a hemodynamic response to the resulting sudden, transient increase in intrathoracic and intra-abdominal pressure. The commonly accepted Valsalva ratio, originally described by Badawa and Ewing, will not differentiate between sympathetic and parasympathetic functions; however, the ratio is used in standardized scoring methods to compare different populations [26–28, 39, 40].

The orthostatic challenge (performed either with the head-up tilt test or by actively standing-up) predominantly evaluates adrenergic function. The 30:15 RRI ratio (the ratio of the HR increase that occurs at approximately 15 s after standing to the relative bradycardia that occurs at approximately 30 s after standing) allows for adrenergic function evaluation, due to vagal withdrawal and sympathetic activation [26–28, 39, 41–43]. Alternatively, a diagnosis of orthostatic hypotension during the tilt test may be used [26–28].

Finally, the isometric challenge measures cardiovagal function, without affecting peripheral vascular resistance. Continuous gripping of a dynamometer at 30% of maximum generates, via lightly myelinated mechanosensitive group III and unmyelinated chemosensitive group IV muscle afferents and the central nervous system, an increase in efferent sympathetic activity [39, 44–47]. The results are reported as the change in diastolic blood pressure (dBP).

### Data extraction and meta-analysis

To ensure sensitivity of analysis, we initially used robust selection criteria. We collected the data into an Excel table, then reviewed the data - assessing each of the extracted articles’ methodology and studied populations. Missing data (i.e., mean deviations, standard errors of mean or standard deviations) were recalculated using the published results available. Assessment of risk of bias was conducted according to Hoy et al. [48]. (Table 1)

**Table 1:** Assessment of risk of bias according to Hoy et al. [46]

References	Year	Assessment criteria of study bias^a^										
		1	2	3	4	5	6	7	8	9	10	11
Boiardi et al. [4]	1988	High	Moderate	Low	Moderate	Low	Low	Low	Low	High	Low	Moderate
Havanka-Kanniainen et al. [51]	1986	Moderate	Moderate	Low	Low	Low	Low	Low	Low	High	Low	Moderate
Havanka-Kanniainen et al. [52]	1986	High	Moderate	Low	Moderate	Low	Low	Low	Low	High	Low	Moderate
Havanka-Kanniainen et al. [53]	1987	Moderate	Moderate	Low	Low	Low	Low	Low	Low	Moderate	Low	Low
Havanka-Kanniainen et al. [54]	1988	Low	Moderate	Low	Moderate	Low	Low	Low	Low	High	Low	Moderate
Pogacnik et al. [56]	1993	Moderate	Moderate	Low	Moderate	Low	Low	Low	Low	High	Low	Moderate
Qavi et al. [57]	2023	Moderate	Moderate	Low	Moderate	Low	Low	Low	Low	High	Low	Moderate

^a^External validity (1–4): (1) Was the study’s target population a close representation of the national population in relation to relevant variables, e.g. age, sex, occupation? (2) Was the sampling frame a true or close representation of the target population? (3) Was some form of random selection used to select the sample OR was a census undertaken? (4): Was the likelihood of non-response bias minimal? Internal validity (5–10): (5) Were data collected directly form the subjects? (6) Was an acceptable case definition used in the study? (7) Was the instrument that measured the parameter of interest shown to have reliability and validity (if necessary)? (8) Was the same mode of data collection used for all subjects? (9) Was the length of the shortest prevalence period for the parameter of interest appropriate? (10) Were numerator(s) and denominator(s) for the parameter of interest appropriate? Summary item on the overall risk of study bias (11): (11) Low risk of bias: further research is very unlikely to change our confidence in the estimate. Moderate risk of bias: further research is likely to have an important impact on our confidence in the estimate and may change the estimate. High risk of bias: further research is very likely to have an important impact on our confidence in the estimate and is likely to change the estimate

The data included in the final analysis was considered as continuous (with average values, standard deviation, standard mean errors) and we analysed the data using the fixed-effects model. The results of fixed-effects analyses are reported, as they provide a more reliable estimate of the true effect [49]. True effect estimates were calculated as adjusted standardized mean differences (Hedge’s g) for deep breathing, isometric challenge and orthostatic challenge results, since these results were represented using different scales. The difference in means was expressed for Valsalva results, since all included articles used the Valsalva ratio. Heterogeneity was assessed using the standard I^2^ index, chi-square, and Tau^2^ tests [50]. We conducted grouped analysis based on the individual ANS tests. We conducted additional subgroup analyses (test of exclusion), to examine the potential for population bias in the Havanka-Kanniainen et al. papers [51–54]. All statistical calculations were performed using RevMan (version 5.4, Copenhagen: The Nordic Cochrane Centre, The Cochrane Collaboration, 2014) [55].

## Results

The systematic review of the literature revealed 679 abstracts (after removing duplicates, *n* = 258), which were published from 1958 to November 2023 and evaluated for eligibility, to be included in the meta-analysis. Search details and reasons for exclusion of studies are shown in Fig. 1. An additional 7 articles were found via citations from the 102 articles. We qualitatively and quantitatively evaluated full text articles for 109 of 686 search results. Eighty-five of the 109 articles did not use Ewing’s suggested autonomic testing protocol [25–28]. A further five did not have healthy controls in their study; one study did not specify the age of the participants; and, another study did not publish the standard differences of the deep breathing and Valsalva values, as well as the R-R interval ratios. Ten articles followed the suggested autonomic testing protocol; however, these were not included due to unattainable additional information required in the final analysis.

### Included studies

Seven of the 109 articles matched our inclusion criteria [4, 51–54, 56, 57]. The articles were published from 1986 to 2023 and included a total of 424 migraine patients and 268 healthy controls. Article characteristics are described in Table 2. The respective interictal deep breathing, Valsalva manoeuvre, orthostatic challenge and isometric challenge results of these seven articles were pooled together. Boiardi and colleagues [4] investigated patients with common migraine interictally; stating that “none of the headache sufferers was tested during a painful attack” [4]. Four studies from Havanka-Kanniainen et al. qualified for the final analysis [51–54]. The initial two 1986-articles from the group examined ANS function in patients with classic migraine and common migraine (with and without aura, respectively) [51, 52]. Differences between the two studies were twofold: the age of the participants (11–22 years [52] and 26–54 years [51], respectively); and timing of ANS function testing, which was performed not only interictally, but also ictally in the latter [51]. The third article of this group [53] evaluated the effects of nimodipine in adult migraine patients using ANS function testing (before and after treatment). The final article from Havanka-Kanniainen and colleagues [54] examined ANS function in a large group of over 180 migraine patients interictally. In all but one article [53], a “headache-free period” of at least five days is described. Pogacnik and colleagues (1993) studied migraine patients with and without aura interictally (“Testing was carried out during the headache free period”) [56]. Qavi and colleagues published the latest findings (2023 print), and studied migraine patients at least 7 days post migraine headache and compared the results with tension-type headache patients and healthy controls [57]. Details on extracted ANS function values and conditions, as well as definitions of the interictal migraine phase, are provided in Table 3.

**Table 2:** Characteristics of studies

Reference	Boiardi et al. [4]	Havanka-Kanniainen et al. [51]	Havanka-Kanniainen et al. [52]	Havanka-Kanniainen et al. [53]	Havanka-Kanniainen et al. [54]	Pogacnik et al. [56]	Qavi et al. [57]
**Year**	1988	1986	1986	1987	1988	1993	2023
**Country**	Italy	Finland	Finland	Finland	Finland	Slovenia	India
**Study design**	Case–control	Case–control	Case–control	Clinical Trial	Case–control	Case–control	Case-control
**N (female), MP/HC**	102 (50), 68/34	20 (18), 10/10	74 (55), 49/25	40 (15), 21/19	273 (192), 188/85	107 (67), 62/45	50 (36), 50/50
**Age mean (SD) MP/HC**	37.9 (1.7)/35.4 (1.6)	41.5 (8.6)/41.4 (4.6)	17.4 (2.8)/17.8 (3.9)	40.8 (8.8)/36.4 (6.8)	30.4 (12.7)/28.3 (11.3)	36.5 (7.6)/35.6 (8.2)	27.7 (8.3)/ 28.3 (8.7)
**Age Range MP/HC**	N/A	24–56/(N/A)	11–22/10–22	21–54/(N/A)	11–69/10–61	21–50/22–49	15–50/15–50
**Diagnosis Criteria**	AHC-CoH	AHC-CoH	AHC-CoH	AHC-CoH	AHC-CoH	IHS	ICHD-3
**Migraine type**	COM	COM & CLM	COM & CLM	COM & CLM	COM & CLM	MwA & MoA	MwA & MoA
**Aura**	Not specified	Not specified	With and without	Not specified	With and without	With and without	With and without
**Attack Frequency**	Not specified	Episodic	Episodic	Episodic	Episodic	Not specified	Episodic
**Other Comorbidities**	None	Not specified	None	None	None	Not specified	None
**Therapy specified**	Not specified	None	None	Nimodipine	None	None	None

MP – Migraine patients; HC – Healthy controls; SD – Standard deviation; N/A – not made available; AHC-CoH – Ad Hoc Committee for Classification of Headache; IHS – International Headache Society; COM – Common migraine; CLM – Classic migraine; MwA – Migraine with aura; MoA – Migraine without aura

**Table 3:** ANS function values and conditions

Reference	Boiardi et al. [4]	Havanka-Kanniainen et al. [51]	Havanka-Kanniainen et al. [52]	Havanka-Kanniainen et al. [53]	Havanka-Kanniainen et al. [54]	Pogacnik et al. [56]	Qavi et al. [57]
**Measurement**	Interictal	Interictal**	Interictal	Not specified***	Interictal	Interictal	Interictal
**Deep Breathing** ^**A**^	20.9 ± 7.9	1.4 ± 0.2	1.5 ± 0.2	1.3 ± 0.1	1.4 ± 0.2	1.5 ± 0.2	27.5 ± 11.0
**Valsalva Manouvre** ^**B**^	2.1 ± 2.7	1.5 ± 0.3	1.7 ± 0.4	1.5 ± 0.3	1.7 ± 0.4	1.9 ± 0.4	1.4 ± 0.4
**Orthostatic Challenge** ^**C**^	1.2 ± 0.0	1.3 ± 0.2	1.4 ± 0.2	1.3 ± 0.16	1.3 ± 0.2	1.6 ± 0.2	1.0 ± 0.1
**Isometric Challenge** ^**D**^	15.7 ± 6.6	17.0 ± 6.0	5.2 ± 8.6	15.2 ± 9.4	16.4 ± 10.2	7.2 ± 7.1	15.6 ± 12.4

* - Data represented as standard deviation after converted from standard error of the mean using formula SD = SE x √(n)

** - Study measured interictal and ictal ANS function values; meta-analysis was conducted using the interictal data

*** - Study cited measurement methods of previous studies, interictal ANS function measurements were assumed

^A^ – Deep breathing: Boiardi et al. and Qavi et al. reported: Mean Difference Minimum-Maximum; other included studies reported: RR-Interval Variation Ratio

^B^ – Valsalva manoeuvre: all studies reported: Valsalva ratio

^C^ – Orthostatic challenge: Boiardi et al., Pogacnik et al. & Qavi et al. reported: RR-Interval 30:15 ratio; Havanka-Kanniainen et al. reported: RR-Interval variation ratio

^D^ – Isometric challenge: Boiardi et al. reported: Mean Difference dBP; Pogacnik et al. reported: Handgrip ratio dBP; Havanka-Kanniainen et al. & Qavi et al. reported: maximum change dBP

### Effects of meta-analysis

**Fig. 2:**
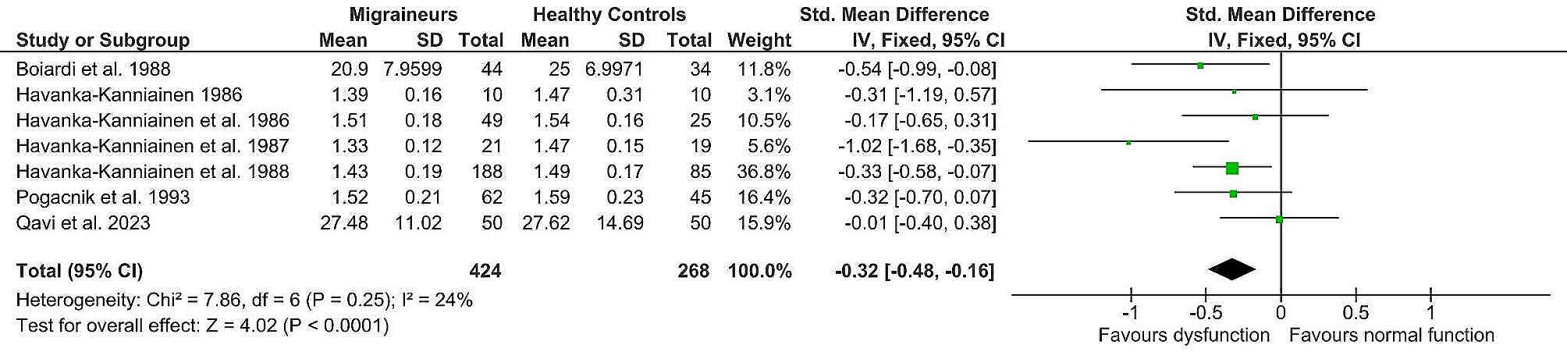
Fixed-effect meta-analysis main effect Forrest plot of deep breathing values (expressed in different scales); where left of 0 favours cardiovagal dysfunction and right of 0 favours normal cardiovagal function

**Fig. 3:**
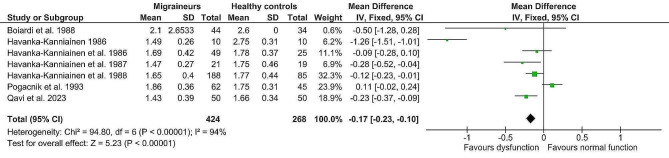
Fixed-effect meta-analysis main effect Forrest plot of Valsalva manoeuvre (expressed as Valsalva ratio); where left of 0 favours sympathetic adrenergic and cardiovagal dysfunction and right of 0 favours normal sympathetic adrenergic and cardiovagal function

**Fig. 4:**
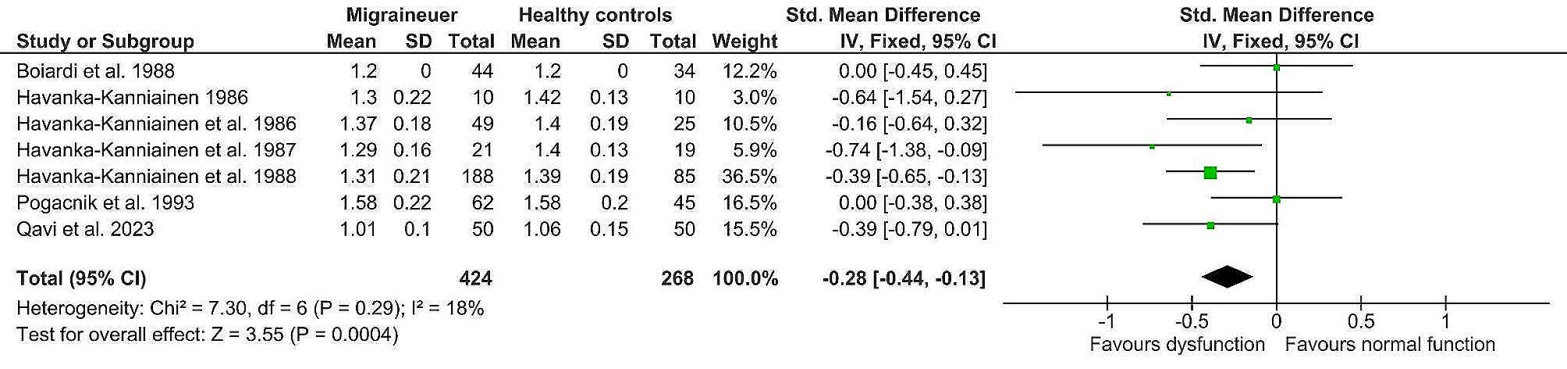
Fixed-effect meta-analysis main effect Forrest plot of orthostatic challenge (expressed in different scales); where left of 0 favours adrenergic dysfunction and right of 0 favours normal adrenergic function

**Fig. 5:**
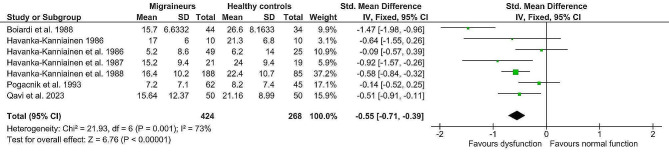
Fixed-effect meta-analysis main effect Forrest plot of isometric challenge (expressed in different scales); where left of 0 favours sympathetic dysfunction and right of 0 favours normal sympathetic function

The fixed-effect analysis of the individual methods (deep breathing, Valsalva manoeuvre, orthostatic challenge, isometric challenge) displayed significantly lower values in the migraine population (*n* = 424) compared to healthy controls (*n* = 268). (Table 4) Lower deep breathing results (mean difference minimum-maximum and R-R interval variation ratio, Z = 4.02, p = < 0.0001, g= -0.32; 95% confidence interval (CI: -0.48, -0.16; k = 7) indicated lower interictal cardiovagal activity in migraine patients. Lower Valsalva manoeuvre results (Valsalva ratio, Z = 5.23, p = < 0.0001, mean difference (MD) = -0.17; 95% CI: -0.23, -0.10) indicated impaired interictal sympathetic adrenergic and cardiovagal functions. Furthermore, lower orthostatic challenge results (R-R Interval 30:15 ratio; R-R Interval variation ratio, Z = 3.55, *p* = 0.0004, g= -0.28; 95% CI: -0.44, -0.13) suggested lower interictal adrenergic function in migraine patients. And finally, lower aggregated isometric challenge results (mean difference dBP; handgrip ratio dBP; and maximum change dBP, Z = 6.76, p = < 0.00001; g= -0.55; 95% CI: -0.71, -0.39) further indicated lower interictal sympathetic function in migraine patients compared to healthy controls. Details are shown in Figs. 2, 3, 4 and 5. The heterogeneity was low for deep breathing and orthostatic challenge (I^2^ = 24% and I^2^ = 18%, respectively) and relatively high for isometric challenge and Valsalva manoeuvre (I^2^ = 73% and I^2^ = 94%, respectively). Despite heterogeneity being relatively high across the studies, the overall effects of meta-analysis were still statistically significant when random-effects analysis was applied. For deep breathing the Z-value was 3.41, *p* = 0.0006. The Z for Valsalva ratio was 2.26 (*p* = 0.02), for orthostatic challenge the Z was 3.02 (*p* = 0.002), while the Z of isometric challenge was also notably decreased to 3.52 (*p* = 0.0004). As such, a random-effects model also indicated lower autonomic function scores in migraine patients compared to healthy controls. A test of asymmetry was not performed, as less than ten studies qualified for the final analysis.

**Table 4:** Data and analyses

Outcome or Subgroup	Studies	Participants	Statistical Method	Effect Estimate
Deep Breathing	7	692	Std. Mean Difference (IV, Fixed, 95% CI)	-0.32 [-0.48, -0.16]
Valsalva Ratio	7	692	Mean Difference (IV, Fixed, 95% CI)	-0.17 [-0.23, -0.10]
Orthostatic Challenge	7	692	Std. Mean Difference (IV, Fixed, 95% CI)	-0.28 [-0.44, -0.13]
Isometric Challenge	7	692	Std. Mean Difference (IV, Fixed, 95% CI)	-0.55 [-0.71, -0.39]

Migraine patients vs. Healthy Controls; IV – Inverse variance; CI – Confidence interval

### Risk of bias in included studies

**Fig. 6:**
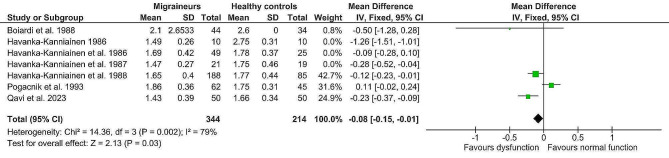
Fixed-effect meta-analysis main effect Forest plot of comparison: Valsalva Ratio with the Havanka-Kanniainen et al. 1986–1987 articles removed from analysis

The results of bias analysis can be seen in Table 1. A high risk of bias was identified with regards to population age in the Boiardi et al. [4] and first of the Havanka-Kanniainen et al. articles [52]. Furthermore, the length of the shortest prevalence period for the parameter of interest presented high risk of bias in all but one study. All but one of the studies observed the headache-free or interictal period, and the peri-ictal ANS function values may differ even more significantly from those of healthy controls, based on findings from Havanka-Kanniainen et al. [51]. Reporting bias was additionally controlled for using strict inclusion criteria and by inspection of heterogeneity. Heterogeneity was assessed using the standard I^2^ index, chi-square, and Tau^2^ tests and by visual inspection. A fixed-effects model was employed, since the analysed data was obtained using the same examination methods, with the same disease population. The resulting statistical heterogeneity was expected, considering that clinical and methodological diversity always occur in a meta-analysis [50]. Finally, bias was examined using a funnel plot of effect size against standard error for asymmetry. Lastly, population bias within the Havanka-Kanniainen et al. articles [51–54] showed changes in the effects sizes, most readily seen in the Valsalva manoeuvre results (Fig. 6); thus, allowing us to conclude – although not definitely – that the same population was not used for the group’s final paper [54].

## Discussion

The present meta-analysis shows that interictal differences in ANS function can be observed in migraine patients compared to healthy controls – that is, all ANS function test values were found to be significantly lower in migraine patients. Meta-analysis revealed a significant main effect with respect to sympathetic adrenergic function (MD_Valsalva manoeuvre_ = -0.17; Hodge’s g_orthostatic challenge_ = -0.28; Hodge’s g_isometric challenge_ = -0.55) in migraine patients – implying that the sympathetic and baroreceptor signalling in these patients was disrupted compared to their healthy peers. Furthermore, a larger main effect was shown for cardiovagal function (Hodge’s g_deep breathing_ = -0.32; MD_Valsalva manoeuvre_ = -0.17); involving the vagal nerve in the manifestation of migraine episodes [25–28]. Considered together, the data suggest that ANS homeostasis in migraine patients is lower – compared to healthy individuals – reacting to changes in ANS signalling levels (such as CGRP) with increased sensitivity.

This phenomenon may be due to the increased quantities of circulating autonomic signalling molecules such as CGRP [58–61]. CGRP’s effects outside of the blood-brain-barrier (BBB) have been well documented [14–19]; however, within the BBB (that is, centrally) there is room for discussion. The mere fact that “CGRP and/or its receptor have been found in the cortex, hippocampus, thalamus, hypothalamus, pituitary, striatum, amygdala, cerebellum, and such migraine-relevant sites in the brainstem as the locus ceruleus, raphe nuclei, and the trigeminal nucleus caudalis” [20] shows the remaining potential to learn about migraine’s pathophysiology. From an ANS perspective, many of these neuroanatomical sites correlate with the autonomic central network [62]. We are unfortunately limited to speculation at this point, with respect to addressing “cause-and-effect”, as CGRP’s half-life causes sampling difficulties peripherally [63–67] – while CSF testing by means of lumbar puncture has not yet been published. This makes correlating CGRP levels and autonomic function very difficult. Moreover, the paroxysmal autonomic symptomatology, which manifests during peri-ictal migraine phase of the cycling episodes [68], may represent a point below which ANS function drops – possibly due to CGRP overproduction inherent to the migraine phenotype or possibly due to overproduction or overflow of other neurotransmitters [58, 59, 69–74]. Consequently, this overflow may tip the nervous system into the well-described pre-ictal, ictal and post-ictal phases of migraine [75–77].

These findings bring into question the roles of other prophylactic migraine medication and its influence on the ANS of migraine patients. One possible explanation could be that, due to their lipophilicity, beta-blockers (e.g. propranolol, bisoprolol and metoprolol) [78, 79] could actually contribute to antagonization of other adrenergic and noradrenergic signalling pathways of the central autonomic network (such as in the insular cortex) [80]. Further pharmacological effects of beta-blockers and angiotensin antagonists [81], in terms of their prophylactic roles, was not investigated with respect to the ANS, in the available literature. Controlled migraine trials focused relatively miopically on outcomes such as headache-free days or acute-medication consumption, without necessarily accounting for all of the symptoms which accompany migraine - autonomic symptoms such as drowsiness, nausea, or changes in appetite [77]. Collecting information regarding autonomic symptoms in migraine should further advance our understanding of this disease as a whole.

Our systematic review identified two articles which conducted their ANS testing during the ictal phase of migraine [51, 82]. These found no statistically significant difference between the ictal and interictal values; however, the ictal and healthy control values differed statistically in one of the articles [51]. We hypothesize, based on the data available [51, 82], that ANS function in the peri-ictal phase of migraine may be even lower than the aggregated values reported in our meta-analysis of interictal data.

It is relevant to note that the included ANS function values (with one exception [57]) were initially measured in the late 1980s and early 1990s [4, 51–54, 56], when the “autonomic theory” of the pathophysiology of migraine was among the main hypotheses suggested to explain migraine. As the theory was disproven, these ANS function values remained unaccounted for. The discovery, as well as clarification of the physiological roles, of CGRP and other relevant neurotransmitters (such as pituitary adenylate cyclase-activating polypeptide (PACAP), glyceryl trinitrate (producing nitric oxide), etc.) [58, 59, 69–74] allowed researchers to correlate neurotransmitter levels with ANS dysfunction in migraine. Furtherstill, a genome-wide association study of migraine patients found 38 distinct genome loci associated with 44 independent susceptibility markers for forms of migraine [83]. Among these was the NGF gene (nerve growth factor) which was shown to be associated with hereditary sensory and autonomic neuropathy, type 5 [84]. Many of the other loci identified have roles either in the structures of the brain where ANS signalling takes place or in human vasculature. In summation, ANS function testing may have a new supporting role as a biomarker of migraine.

### Agreements and disagreements with other studies or reviews

Three recent autonomic function, case-control studies were not included in our final analysis due to a lack of data; which were unattainable after attempting to contact the corresponding authors [36–38]. All three studies were conducted after Novak published the standardized version of the ANS testing protocol [28]. One of these studies postulated that there exists an impairment of the primary autonomic system and/or neurotransmitter function in migraine patients [38]. Meanwhile the other two studies suggested that there exists an increased vasomotor reactivity in patients with migraine [36, 37]. These conclusions would appear to agree with the data we aggregated. Other studies looked at autonomic function in migraine patients, either with isolated autonomic tests (exclusive HRV analysis through electrocardiography - ECG) or parts of ANS function testing protocols (HRV using the head-up tilt-table test). Miglis [15] comprehensively reviewed these ANS investigations conducted in migraine patients – therefore, it was not the aim of this paper to repeat his findings. Rather, we aimed to supplement his work, by accumulating the published ANS values, albeit, for individual tests most similar to the internationally accepted quantitative autonomic testing protocol described by Novak [28].

Searching Pubmed for meta-analyses investigating ANS function and migraine produced only a handful of results. Of these, none analysed publications which used the protocols suggested by Ewing et al. or Novak [25–28]. The meta-analysis by Lee et al. looked at ECG findings in migraine patients. The initial problem in this study is that two ECG recording methods (24-hour ambulatory vs. short duration) were analysed together – yielding different amounts of autonomic data for analysis [29]. Further still, the authors analysed certain cardiac autonomic results, excluding other results describing autonomic function in the studied populations (i.e. not using tilt-table test values from the Mosek et al. study [85]). The meta-analysis by Koenig et al. aimed to analyse the HRV in headache patients vs. controls – using various methodology to arrive at HRV results. While HRV is the beat-to-beat variation of heart rate, the methods ranged from measurements over five minutes to those over 48 h [9]. Therefore, there currently exist no meta-analyses which summarize ANS function data gathered using the standardized ANS testing protocol.

### Potential biases in the review process

Our systematic review faced several potential limitations. We employed a specific set of criteria, to reduce bias; however, these criteria limited the publications which were included – namely, from only three research groups. Additional publications met the inclusion criteria [36–38, 82, 85–90]; however, these did not report the required values and the corresponding authors were unreachable, so that the missing information could be obtained. The meta-analysis reviewed studies which measured the ANS function parameters, examined in the latest guidelines to autonomic testing [28]. Unfortunately, none of the included studies followed these guidelines, nor used the composite autonomic severity score. Furthermore, a high risk of bias (Table 1) could be seen in all but one study, as the peri-ictal ANS function values may differ even more significantly from those of healthy controls, with respect to the length of the shortest prevalence period for the parameter of interest [53]. That is, interictal ANS testing was conducted once per patient and there exists a high chance that ANS functions may differ when averaged throughout an entire month. Furthermore, perhaps the ictal measurements also differ in relation to when in the migraine cycle, the ANS testing was conducted (pre-ictal vs. ictal vs. post-ictal).

Importantly, the articles published by Havanka-Kanniainen et al. [51–54] did not disclose whether the same study population was used throughout their publications. They cite their previous studies [51–53] in the final article [54]; allowing us to believe that the data in the final article is original. A test of exclusion found that the three articles did not uniformly affect the significance of the individual tests; moreover, only Valsalva ratio was shown to cross the zero-line upon exclusion of the earlier three results. (Fig. 6) An additional argument for inclusion of all four articles is that the final article [54] summarized ANS function in 273 migraine patients interictally, while the other articles [51–53] investigated other hypotheses.

### Quality of the evidence

The body of evidence concerning ANS function testing in migraine patients is not negligible; however, the structure with which it was conducted (i.e., methodology, reported results) is heterogeneous. We were able to include seven articles, although an additional ten qualified based on respective methodologies [36–38, 82, 85–90]. Of the seven articles included, the biggest variation – and thus limitation – was in the results reported. For example, for the isometric challenge, one group reported the mean difference in diastolic blood pressure [4], the second group reported the maximum change in diastolic blood pressure [51–54], while the third group decided to measure “the average R-R interval during the 15 seconds preceding the contraction … divided by the minimal R-R interval during the contraction period” [56]. The last group decided to measure BP “before the grip and at the one-minute intervals during handgrip“ [57]. All four of these variations are correlates of cardiovagal function, but exemplify the inconsistency of autonomic testing at its infancy. Moreover, the meta-analysis of these data required calculating the standard mean differences, due to the inconsistency in scales used by the individual groups. Therefore, this is a large limiting factor of the results published at this time and, by extension, of our meta-analysis.

### Overall completeness and applicability of evidence

This meta-analysis offers a complete and systematic overview of the published ANS function tests which are relevant to examine in migraine patients. The paper presents the values expected in this patient population. In the composite autonomic severity score (CASS), initially suggested by Low [26], sudomotor function testing is also one of the three main components. This, however, was not initially part of Ewing’s suggested testing methodology [25] and, therefore, it was impossible to conduct a meta-analysis using the CASS. Moreover, articles citing the latest autonomic testing protocol (later than Novak’s 2011 article [28]) in their methodology, did not include all the values required for our meta-analysis nor sudomotor function results [36–38].

## Conclusion

This systematic review and meta-analysis shows – with the limited data available – that ANS function is significantly impaired in migraine patients. The ANS values included in this meta-analysis were gathered during the interictal phase of the patients’ migraine cycles – more precisely, without paroxysmal autonomic symptoms associated with the peri-ictal migraine phase. The data suggest, ANS function in migraine patients operates at a lower threshold of homeostasis during the interictal phase of the migraine cycle.

### Implications for Methodological Research

The impact of autonomic migraine symptoms – as well as increased likelihoods of cardio- and cerebrovascular events – go underappreciated in daily clinical practice. The aggregated results from the meta-analysis allow future research questions to have a reference for ANS function in the migraine population.

Even though autonomic nervous system dysfunction cannot lead to migraine diagnosis, more attention on ANS dysfunction may help to further elucidate its role as a biomarker of migraine and improve the management of migraine patients. Future research using smartphone headache diaries would also benefit from gathering the autonomic prodromal symptom data, to build upon our presented findings and further elucidate the pathophysiology of individual migraine attack. This should help establish earlier warning signs, which ultimately can benefit patient guidance, regarding administration of abortive migraine medication – such as triptans – which show greater effect when administered earlier in the migraine attack phase. Additionally, ANS testing offers an extra method with which researchers can quantify the effect of increased presence of CGRP – or perhaps other neurotransmitters – found in migraine patients [14, 16, 58–61, 69–74, 91, 92].

In light of the growing use and effectiveness of anti-CGRP-mAb therapy, this meta-analysis should offer a foundation upon which further ANS function research – as well as clinical trial research – can create future experimental methodologies, which more closely observe (in addition to the standardized side-effect and severe adverse event reporting) the effects of these new and rapidly developing therapies.

Contributions of Authors.

ARP and CW conceived the study and developed the protocol with KZ. ARP was responsible for data collection and statistical analysis supported by KZ and CW. The manuscript was drafted by ARP and revised by KZ and CW. All authors approved the final version.

## Data Availability

Data supporting the findings of this study are available from the corresponding author upon reasonable request by a qualified researcher and upon approval by the data-clearing committee of the Medical University Vienna.

## Appendix

A—search strategy by database as of November 2023 PubMed: (((autonomic testing) OR (autonomic function)) AND (migraine)) NOT (review): 672 hits; Cochrane Database for Systematic Reviews and Cochrane Central Register of Controlled Trials: (((autonomic testing) OR (autonomic function)) AND (migraine)) NOT (review): 11 hits; CINAHL: (autonomic testing) OR (autonomic function) AND (migraine) NOT (review): 17 hits; Web of Science: (((autonomic testing) OR (autonomic function)) AND (migraine)) NOT (review): 237 hits.

## Abbreviations

ANS: autonomic nervous system
PRISMA: Preferred Reporting Items for Systematic Reviews and Meta-Analyses
MOOSE: Meta-analyses of Observational Studies in Epidemiology
CI: confidence interval
g: the standardized mean difference
MD: difference of means
CGRP: calcitonin gene-related peptide
CVD: cardiovascular disease
HR: hazard ratio
OR: odds ratio
HRV: heart rate variability
CASS: composite autonomic scoring scale
CINHAL: Cumulative Index to Nursing and Allied Health Literature
ICHD: International Classification of Headache Disorders
AHC-CoH: Ad Hoc Committee for classification of migraines
RRI: R-R-Interval
E: I ratio: expiratory-inspiratory ratio
dBP: diastolic blood pressure
PACAP: pituitary adenylate cyclase-activating polypeptide
NGF: nerve growth factor
ECG: electrocardiography
